# Difficult airway simulation-based training for anaesthesiologists: efficacy and skills retention within six months

**DOI:** 10.1186/s12871-024-02423-x

**Published:** 2024-01-31

**Authors:** Kateryna Bielka, Iurii Kuchyn, Hanna Fomina, Olena Khomenko, Iryna Kyselova, Michael Frank

**Affiliations:** 1 Postgraduate department of Surgery, Anaesthesiology and Intensive Care, Bogomolets National Medical University, Kyiv, 01601 Ukraine; 2 Shupyk National Healthcare University of Ukraine, Kyiv, Ukraine

**Keywords:** Simulation, Difficult airways, Intubation

## Abstract

**Background:**

The aim of this study was to evaluate how anaesthesiologists manage a “cannot intubate, can ventilate” (CI) and “cannot intubate, cannot ventilate” (CICV) scenarios, and how following simulation training will affect their guideline adherence, skills and decision-making immediately after training and 6 months later.

**Methods:**

A prospective controlled study was conducted from July to December 2022. Anaesthesiologists who applied for the continuous medical education course “Difficult Airway Management” were involved in the study. Each volunteer participated in two simulation scenarios (CI, CICV) with structural debriefing after each scenario. After the first simulation round, volunteers were trained in difficult airway management according to DAS guidelines, using the same equipment as during the simulation. The participants repeated the simulation scenarios the day after the training and six months later. The primary and secondary endpoints were compared between three rounds: initial simulation (Group 1), immediately after training (Group 2), and six months after training (Group 3).

**Results:**

A total of 24 anaesthesiologists consented to participate in the study and completed the initial survey form. During the first session, 83.3% of participants had at least one major deviation from the DAS protocol. During the first CICV scenario, 79% of participants made at least one deviation from the DAS protocol. The second time after simulation training, significantly better results were achieved: the number of anaesthesiologists, who attempted more than 3 laryngoscopies decreased (OR = 7 [1.8–26.8], *p* = 0.006 right after training and OR = 3.9 [1.06–14.4], *p* = 0.035 6 month later); the number, who skipped the supralaryngeal device attempt, call for help and failure to initiate surgical airway also decreased. Simulation training also significantly decreases the time to call for help, cricothyroidotomy initiation time, and mean desaturation time and increases the odds ratio of successful cricothyroidotomy (OR 0.02 [0.003–0.14], *p* < 0.0001 right after training and OR = OR 0.02 [0.003–0.16] 6 months after training).

**Conclusions:**

Anaesthesiologists usually display major deviations from DAS guidelines while managing CI and CICV scenarios. Simulation training improves their guideline adherence, skills, and decision-making when repeating the simulation immediately after training and 6 months later.

**Study registration:**

NCT05913492, clinicaltrials.gov, 22/06/2023.

## Introduction

Difficult airways remain a significant problem in anaesthesia, intensive care, and emergency medicine. “Сannot intubate, cannot ventilate” situations account for nearly 25% of anaesthetic mortality [1, 2], which makes them the most common lethal respiratory emergency in perioperative settings [3].

Updated recommendations of the Difficult Airway Society (2015) cover unanticipated difficulties in routine intubation and rapid sequence induction [4]. The recommendations suggest planning for failed intubation, meticulous preparation, using algorithms as a standard for every possible situation, appropriate post-event debriefing. In addition, the development of modern technologies and the appearance of new devices require specific knowledge and skills [5]. That creates a demand for difficult airway management training. Simulation-based training gives better outcomes compared to non-simulation and non-intervention education [6]. However, it remains unclear how long the acquired skills are retained and how often simulation training should be repeated.

The aim of this study was to evaluate how anaesthesiologists manage a “cannot intubate, can ventilate” (CI) and “cannot intubate, cannot ventilate” (CICV) scenarios, and how simulation training will affect their guideline adherence, skills and decision-making right after training and 6 months later.

## Materials and methods

A prospective controlled study was conducted at the Postgraduate Department of Surgery, Anaesthesiology, and Intensive Therapy at Bogomolets National Medical University from July to December 2022. The study design was approved by Bogomolets National Medical University ethical committee (protocol #148, 07.09.2021) and retrospectively registered at clinicaltrials.gov (NCT05913492, 12/6/2023). Anaesthesiologists who applied for the continuous medical education course “Difficult Airway Management” were involved in the study. The course was conducted in Kyiv and some of the participants have traveled from other regions of Ukraine to attend it. Information on their current employment and university education was not recorded. None of the participants were employed at the study site. We obtained consent from them to participate anonymously as volunteers. Before the initial training, all participants were interviewed about their experience working in the speciality, difficult airway management, learning at any simulation training, and difficult airway training.

The simulation room included a Laerdal SimMom mannequin Advanced Patient Simulator, a vital monitor, a LEON anaesthesia station, and airway devices. Standard settings included modelling of tongue edema to grade 4 Cormack and Lehane visualizations on laryngoscopy and additional pharyngeal obstruction and stiffness of both lungs for the “cannot ventilate” scenario. A set of tools and equipment, sufficient for fulfilment of every step of the DAS protocol was provided. Instruments for ensuring airway patency, ventilation, and tracheal intubation included facemasks, oropharyngeal and nasal airways, laryngoscopes, laryngeal masks, and tracheal tubes in various sizes. The trolley for difficult airways was available at each station and was equipped with additional laryngoscope blades of various sizes, a video laryngoscope, laryngeal masks of multiple sizes (I-gel), introducers for tracheal tubes (stylets and bougies), Airtraq, cricothyroidotomy kit.

Monitoring provided were SpO2, EtCO2, ECG, and non-invasive blood pressure measurements. When the oxygen delivery was interrupted for 20 s or more, the SpO2 gradually decreased by 3% every 5 s and reached 90% after 20 s. A value of SpO2 < 90% was considered desaturation. Adequate ventilation was defined as at least two effective breaths, evidenced by an EtCO2 curve on the monitor, which was digitally simulated and controlled through instructor input, according to the conditions of the given scenario. Every volunteer went through two simulation scenarios of difficult airway management: (1) “cannot intubate, can ventilate” (CI), (2) “cannot intubate, cannot ventilate” (CICV) with structured debrief after both scenarios. Participants were expected to follow the algorithm according to the Difficult Airway Society (DAS) recommendations [4]. The standardized feedback was given with the TALK system: (1) Target: share your perspective, how you filled during the scenario; (2) Analysis: what happened with patient. What you’ve done well, what you could improve next time; (3) Learning points: what can the team learn from that experience; (4) Key actions: home messages, what we’ve learn from the scenario.

During the scenario, we recorded significant deviations from the DAS protocol and other indicators that impacted the quality of the algorithm adherence: scenario instructors filled in the check-list for each scenario. The primary endpoints included significant deviations from the DAS protocol: more than three laryngoscopy attempts; supraglottic airway attempt omitted; call for help omission; failure to initiate a surgical airway (for the CICV scenario). Secondary endpoints included: time to call for help; mean duration of desaturation; use of bougie; use of video laryngoscope (Airtraq); mean number of intubation attempts; improper usage of equipment, time to initiation of the surgical airway; successful cricothyroidotomy incidence. Cricothyrotomy was considered successful when followed by a visible distension of the imitated lung on forced inspiration. “Improper usage of equipment” was defined as ineffective or overly traumatic application of an instrument that has resulted from a faulty technique, as ultimately judged by the observing instructor.

After the first simulation round and structured debrief, volunteers were trained in difficult airway management according to DAS guidelines, using the same equipment as during the simulation. Training include DAS protocol learning, practical work on the SimMom Mannikin (intubation with bougie, Airtraq, cricothyroidotomy with scalpel, bougie, tube technique). The participants repeated the simulation scenarios the day after the training and six months later. The primary and secondary endpoints were compared between three rounds: initial simulation (Group 1), immediately after training (Group 2), and six months after training (Group 3).

Sample size calculation was based on a previous study involving simulators [7, 8] and determined by the number of continuing medical education course “Difficult airway management” attendees who consented to participate in the study.

### Statistical analysis

Demographic and observed performance parameters were compared between the three groups. Normality was checked for all variables with the Kolmogorov-Smirnov test. Normally distributed numerical data are presented as mean ± standard deviation (SD), abnormal distributed data are presented as medians with 25–75% interquartile ranges (IQRs), categorical data are presented as proportions. To assess significance levels, Student’s test was used for normally distributed data, Kruskal–Walli’s test was used for abnormal distributed data, exact Fisher’s test for categorical data, with odds ratio (OR) calculations. The difference was considered significant at α < 5% (*p* < 0.05). We assumed that nearly 70% of the participants would make at least one major deviation from the DAS recommendation during the first scenario. A sample size of 24 would provide 60% power to detect a minimum of 30% decrease in the proportion of participants committing major deviations from the first to the second session, assuming an α error probability of 0.05.

## Results

A total of 24 anaesthesiologists consented to participate in the study and completed the initial survey form. The mean age of the subjects was 35.7 ± 6.5 years in Group 1 and Group 2, and 34.6 ± 5.6 in Group 3. The work experience after an internship was 10.3 ± 5.4 in Group 1 and Group 2 and 9.5 ± 5 years in Group 3. Almost all subjects had experience with difficult airways during their medical careers (cannot intubate, cannot ventilate). It is also notable that seven people in each of the three groups had previous ALS training. None of the doctors had previous experience with cricothyroidotomy. More detailed information about the demographic data of the groups of subjects and their work and training experience are given in Table 1.

**Table 1:** Demographic data of volunteers and their previous experience. Standard deviation (SD) is presented alongside means

	Group 1 (*n* = 24)	Group 2 (*n* = 24)	Group 3 (*n* = 19)
Gender, f (%)	14 (58.3)	14 (58.3)	12 (63.1)
Age (Mean ± SD)	35.7 ± 6.5	35.7 ± 6.5	34.6 ± 5.61
Years of work after internship (Mean ± SD)	10.3 ± 5.4	10.3 ± 5.4	9.5 ± 52
Work experience < 3 years, n	1	1	1
Work experience of 3–9 years, n	10	10	8^1^
Work experience > 10 years, n	13	13	10^1^
Preliminary ALS training, n	7	7	7
Previous experience in simulation training*, n	2	2	12
Previous clinical experience with a difficult airway (cannot intubate can ventilate), n	23	23	18^1^
Previous clinical experience with a difficult airway (cannot intubate cannot ventilate), n	8	8	7^1^
Non-simulated cricothyroidotomy experience, n	0	0	0

^1^*p* > 0.05 Fisher’s test

* Excluding the simulations related to the study

**Fig. 1:**
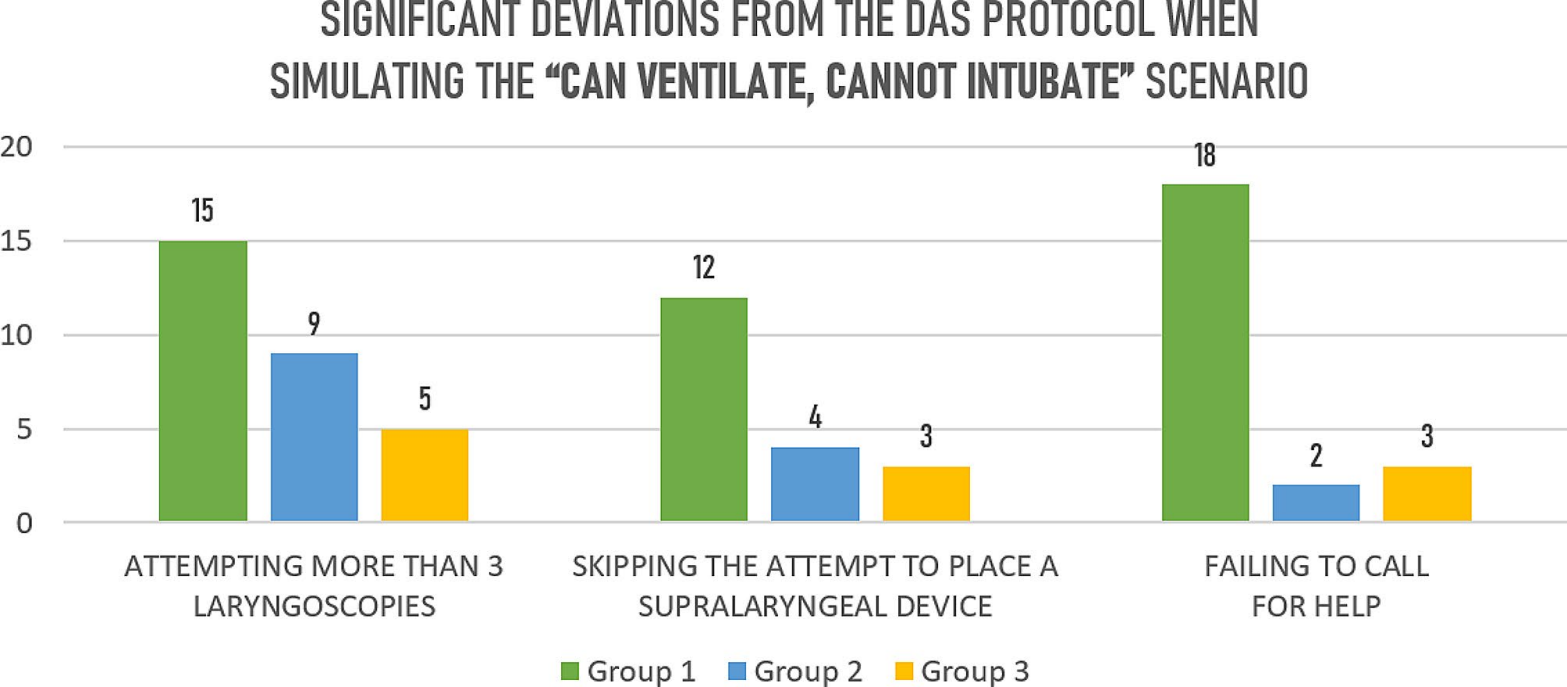
Significant deviations from the DAS protocol during the first scenario, n of cases

During the first session, 83.3% of participants had at least one major deviation from the DAS protocol. The following deviations were recorded: more than three laryngoscopies, skipping attempts to use supraglottic devices, and skipping call for help appeared markedly more often in Group 1 in comparison to Group 2 and Group 3 (Table 2; Fig. 1).

**Table 2:** Significant deviations from the DAS protocol during the CI scenario

Significant deviations from the DAS protocol	Group 1 (*n* = 24)	Group 2 (*n* = 24)	Group 3 (*n* = 19)
More than 3 laryngoscopy attempts, n	15	9^1^	5^1^
Odds ratio, OR [95CI], *p*-value		OR 5.27 [1.5–18], *p* = 0.009	OR 4.6 [1.2–17.3], *p* = 0.03
Supraglottic airway attempt missing, n	12	4^1^	3^1^
Odds ratio (OR)		OR 5 [1.3–19], *p* = 0.03	OR 5.3 [1.2–23.2], *p* = 0.03
Failing to call for help, n	18	2	3
Odds ratio, OR [95CI], *p*-value		OR 33 [95CI 5.9-183.7], *p* < 0.0001	OR 16 [3.4–74.7], *p* = 0.0001

^1^*p* < 0.05

The mean intubation attempts number decreased after training from 4.3 ± 1 in Group 1 to 2.6 ± 0.49 and 2.8 ± 0.38 in Groups 2 and 3 respectively, indicating that anaesthesiologists proceed quicker to plan B or C after the simulation training and these skills were retained after 6 months, moreover they recognized that they would need help more quickly (Table 3). Simulation training also improves anaesthesiologists’ use of additional devices for airway management - bougie (9.2 [2.5–34.6], *p* = 0.001) and Airtraq (18 [3.5–97], *p* = 0.0002) and decreased inappropriate equipment usage. 6 months later, doctors still used these devices more often than before training (OR = 8.2 [2-32.7], *p* = 0.002 and OR = 12 [2.2–67.2], *p* = 0.002 for bougie and Airtraq respectively).

**Table 3:** Other results of CI scenario

Other results	Group 1 (*n* = 24)	Group 2 (*n* = 24)	Group 3 (*n* = 19)
Time to call for help, s	115 ± 10^1^	93.5 ± 1.87	95.9 ± 2.5
The mean duration of desaturation < 90%, s	110 ± 29.5	90.7 ± 6.18	93.4 ± 7.3
Use of bougie, n (%)	5(21)	17(71)	13(68)
Odds ratio, OR [95CI], *p*-value		9.2 [2.5–34.6], *p* = 0.001^2^	8.2 [2-32.7], *p* = 0.002^2^
Use of Airtraq, n (%)	2(8)	15(63)	10(53)
Odds ratio, OR [95CI], *p*-value		18 [3.5–97], *p* = 0.0002^2^	12 [2.2–67.2], *p* = 0.002
Mean number of intubation attempts	4.3 ± 1^1^	2.6 ± 0.49	2.8 ± 0.38
Improper usage of equipment, n (%)	8(33)	1(4)	1(5)
Odds ratio, OR [95CI], *p*-value		11.5 [1.3–101], *p* = 0.022^2^	9 [1–80], *p* = 0.027^2^

^1^*p* < 0.0001, Student’s test; ^2^ Fisher’s exact test

**Fig. 2:**
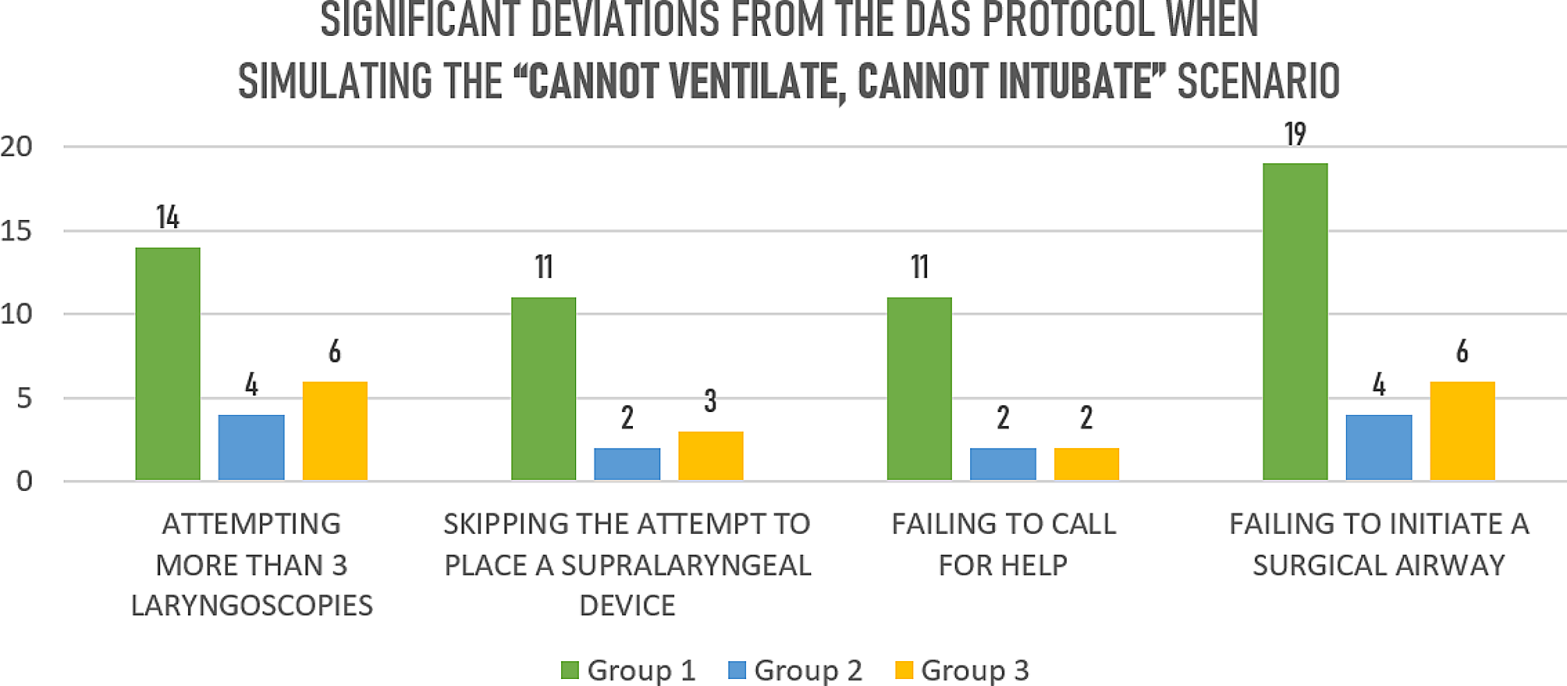
Significant deviations from the DAS protocol in the second scenario, n of cases

During the first CICV scenario 79% of participants made at least one deviation from the DAS protocol. The second time after simulation training, significantly better results were achieved: the number of anaesthesiologists, who attempted more than 3 laryngoscopies decreased (OR = 7 [1.8–26.8], *p* = 0.006 right after training and OR = 3.9 [1.06–14.4], *p* = 0.035 6 month later); also decreased those number, who skipped the supralaryngeal device attempt, call for help and failure to initiate surgical airway (Table 4; Fig. 2). Simulation training has also significantly decreased the time to call for help, cricothyroidotomy initiation time, mean desaturation time and increased the odds ratio of successful cricothyroidotomy (OR 0.02 [0.003–0.14], *p* < 0.0001 right after training and OR = OR 0.02 [0.003–0.16] 6 months after training).

**Table 4:** Significant deviations from the DAS protocol and other results during CICV scenario

Significant deviations from the DAS protocol	Group 1	Group 2	Group 3
Attempted more than 3 laryngoscopies, n (%)	14 (58%)	4 (17%)	6 (32%)
Odds ratio, OR [95CI], *p*-value		7 [1.8–26.8], *p* = 0.006	3.9 [1.06–14.4], *p* = 0.035
Skipping the attempt of supralaryngeal devices, n (%)	11 (46%)	2 (9%)	3 (16%)
Odds ratio, OR [95CI], *p*-value		8.6 [1.7–44.9], *p* = 0.008	4.2 [0.96-18], *p* = 0.058
Skipping a call for help, n (%)	11 (46%)	2 (9%)	2 (11%)
Odds ratio, OR [95CI], *p*-value		8.6 [1.7–44.9], *p* = 0.008	8.6 [1.7–44.9], *p* = 0.008
Failure to initiate surgical airway placement, n (%)	19 (79%)	4 (17%)	6 (32%)
Odds ratio, OR [95CI], *p*-value	-	19 [4.4–81.5], *p* = 0.0003	8 [2-32.7], *p* = 0.002
Other results			
Average time to call for help, s	106.6 ± 10.5^1^	91.2 ± 2.92	91.9 ± 3.5
Time to cricothyroidotomy initiation, s	456 ± 47.8^1^	258 ± 68.7	250 ± 62.2
Median duration of desaturation < 90%, s	180 [160–200]^2^	107 [100–111]	101.5 [96-101.5]
Successful cricothyroidotomy incidence	3/16	18/2	16/2
Odds ratio, OR [95CI], *p*-value	-	OR 0.02 [0.003–0.14], *p* < 0.0001	OR 0.02 [0.003–0.16]
Improper use of equipment, n (%)	9 (38%)	2 (8%)	1 (5%)
	-	6.6 [1.25–34.9], *p* = 0.036	10.8 [1.2–95.2], *p* = 0.026

^1^*p* < 0.0001, Student’s test; ^2^ Kruskal-Wallis test

## Discussion

There are data showing that simulation training is effective in improving anaesthesia residents’ skills in airway management [9]. However, it is not well established if simulation training is also effective for practicing anaesthesiologists, who already have years of experience and preferences on how to manage difficult airways [8].

This is the first study which evaluates the feasibility of the DAS protocol in Ukraine as well as the efficacy of simulation-based training to improve anaesthesiologists’ adherence to DAS guidelines and skill retention 6 months later. Although the study was conducted in a single training centre, the participants were from different regions of Ukraine, which reflects the diverse practice of managing difficult airways in the country.

The first session results outline how experienced anaesthesiologists manage the CI and CICV scenarios. Despite the popularization of DAS guidelines among anaesthesiologists worldwide, nearly 83% of participants had at least one major deviation from the protocol at the CI session. After debriefing with experts during CICV scenario, similarly nearly 80% had at least one deviation. Other authors [8] report similar results, which could be explained by experienced anaesthesiologists’ reluctance to learn new skills and change usual behaviour.

We found significant improvement in adherence to DAS guidelines after simulation training and within 6 months after it. Participants demonstrated fewer deviations from the protocol, calling for help more often whenever a difficult airway scenario was identified and utilizing supraglottic devices more consistently while avoiding ineffective laryngoscopy attempts following three failures. Initial reluctance to initiate surgical airway management was a prominent flaw in the approach of a majority of participants, which was mostly resolved after a single round of simulation training. Duration of significant oxygen desaturation has decreased in both post-training CI and CICV scenarios, which suggests that standardised difficult airway drills may have a positive impact on patient prognosis. This improvement persisted six months after the training. Interestingly, some indicators in Group 3 were even better than in Group 2. For instance, incorrect equipment usage in six months posttraining decreased 6-fold when compared to pre-training and nearly 2-fold compared to immediately after training. Obviously, during these six months, the participants had the opportunity to consolidate the skills while using the equipment correctly in their daily practice.

Most of our results immediately after the course (Group 2) and six months later (Group 3) are comparable to similar studies from other countries [7, 10]. However, in this study it takes longer for anaesthesiologists to call for help - this result may indicate both a shortage of staff in hospitals and a lack of communication and teamwork skills.

In another similar study, the results before and after simulation training did not differ significantly. The authors suggest that multiple factors other than airway algorithms come into play in emergency airway decision-making processes, including one’s personal clinical experience with many available airway devices [8]. At the same time, high cost, technical problems and a variety of approaches limit the widespread use of simulation-based training in low- and middle-income countries [11]. Borges et al. have demonstrated a shortening in time to start cricothyroidotomy and achieve ventilation with no detected positive effect on DAS protocol adherence. We have observed a similar decrease in surgical airway times, which has lasted for at least 6 months and a somewhat less robust improvement in protocol adherence. It is possible, that certain elements of correct difficult airway management are being retained for longer periods, warranting less frequent repetition of costly full simulations, interspaced with instruction in subjects that require multiple sessions to get integrated into clinicians’ routine practice. Recent research comparing low- and high-fidelity difficult airway simulations found the results to be similar, proving that the benefits of simulation training can be made available to anesthesiologists in lower income settings [12].

Experts confirm that assessment in medical education in anaesthesia is a difficult task, especially in airway management [10, 13, 14]. Evaluation during simulation-based training helps to improve the operator’s technical skills, communication with peers in critical situations and patient safety [15–17]. The development of technologies and simulators requires continuous improvement of educational programs and simulation-based assessments [15, 18, 19]. In this situation the creation of standards for simulation became a challenge. Cumin D. and colleagues highlighted that the absence of standards undermines confidence in the results of any simulation-based endeavour and increases the risk of negative learning [16].

A limitation of our paper is the small number of participants, which could influence the study outcome. The majority of the volunteers in our study didn’t have previous experience with mannequin and simulation-based training, which could influence the study results. 8 of 24 initial participants has reported having prior experience with CICV scenarios, which is unusual considering their rarity. It is possible that the group recruited for this study is not fully representative of a general population of anesthesiologists since they could be seeking training due to working in conditions, where such situations are disproportionally common (and have therefore developed local approaches to the problem).

In this study, we have attempted to improve the fidelity of the simulation through employing instructors with extensive background in both anaesthesiology and simulation teaching. Despite this, some delays and distortions cannot be avoided. Simulations of this level of fidelity therefore may not be considered a replacement to the real clinical experience, but are viewed here as a way to complement it. Other issue related to study design itself, as failure to call for help may be influenced by many factors including culture, resources, help available, or being in a simulation where help may not be trusted. Also this factors could influence supraglottic devices use for rescue.

Performance during simulation could possibly be affected by using SimMom instead of a SimMan. In this study we choose SimMom because some scenarios included pregnant patient, and we had this manikin easily available.

Five of the initial participants did not return for the training after 6 months, causing the loss of study power. The reasons for this were not examined. It is possible that some of participants did not find the training useful enough to repeat, did not see the need in repeating it having obtained the expected experience or simply found it inconvenient to travel at the time. Furthermore, we did not have an opportunity to evaluate the difficult airways management skills in 12 months. However, Kuduvalli P. and team in their publication describe a decrease in knowledge and higher deviation from the DAS protocol by anaesthesiologists in one year after mannequin training [7]. Future studies within several educational centres with a higher number of participants and prolonged outcome analysis (skills in one year) can help to understand the specific training requirements of Ukrainian doctors.

## Conclusions

Anaesthesiologists usually display major deviations from DAS guidelines while managing CI and CICV scenarios. Simulation training improves their guideline adherence, skills and decision-making when repeating the simulation right after training and 6 months later.

## Data Availability

The datasets used and/or analysed during the current study are available from the corresponding author on reasonable request.

## Abbreviations

ALS: Advanced life support
CI: Confidence interval
CI: Can’t intubate
CICV: Can’t intubate can’t ventilate
DAS: Difficult airway society
ECG: Electrocardiography
OR: Odds ratio
SAP: Systolic arterial pressure
SD: Standard deviation
SpO2: Peripheral oxygen saturation
